# Accessing HIV Care to Irregular Migrants in Israel, 2019–2024

**DOI:** 10.3390/v17121566

**Published:** 2025-11-29

**Authors:** Tali Wagner, Zohar Mor, Yaakov Dickstein, Dan Turner, Eynat Kedem, Itzchak Levy, Anat Wieder-Finesod, Hila Elinav, Ibrahim Nasser Eddin, Karen Olshtain-Pops, Daniel Elbirt, Rozalia Smolyakov, Valery Istomin, Marina Wax, Yael Gozlan, Orna Mor

**Affiliations:** 1 Department of Tuberculosis and AIDS, Ministry of Health, Jerusalem 9438317, Israel; tali.wagner@moh.gov.il (T.W.);; 2 Faculty of Medicine, Tel-Aviv University, Tel Aviv 6997801, Israel; dant@tlvmc.gov.il (D.T.); itsik.levy@sheba.health.gov.il (I.L.); anat.wieder@sheba.health.gov.il (A.W.-F.); 3 Infectious Disease Unit and HIV Clinics, Rambam Health Care Campus, Haifa 3109601, Israel; y_dickstein@rambam.health.gov.il; 4 Ichilov Medical Center, Crusaid Kobler AIDS Center, Tel Aviv 6423906, Israel; 5 Immunology Unit, Rambam Health Care Campus, Haifa 3109601, Israel; e_kedem@rmc.gov.il; 6 Rappaport Faculty of Medicine, Institute of Technology, Technion, Haifa 3109601, Israel; 7 Infectious Disease Unit, Chaim Sheba Medical Center, Ramat Gan 5262112, Israel; 8 Faculty of Medicine, Hadassah Braun School of Public Health and Community Medicine, Hebrew University, Jerusalem 9112102, Israel; hila.elinav@gmail.com (H.E.); elbirtda@clalit.org.il (D.E.); 9 Hadassah Medical Center, Jerusalem 9112102, Israel; ibrahim.nassereddin@gmail.com (I.N.E.); kerenop@hadassah.org.il (K.O.-P.); 10 Immunology, Kaplan Medical Center, Rehovot 76100, Israel; 11 Goldman Medical School, Faculty of Health Sciences, Ben-Gurion University of the Negev, Beersheba 8410501, Israel; smalikov@bgu.ac.il; 12 Infectious Disease Institute, Soroka Medical Center, Beersheba 84101, Israel; 13 Hillel Yaffe Medical Center, Hadera 38100, Israel; valeryi@hy.health.gov.il; 14 National HIV-1 and Viral Hepatitis Reference Laboratory, Chaim Sheba Medical Center, Ramat Gan 5262112, Israel; marina.wax@sheba.health.gov.il (M.W.); yael.gozlan@sheba.health.gov.il (Y.G.)

**Keywords:** HIV-1 (human immunodeficiency virus), irregular migrants (IMs), public–private partnership (PPP) program, ministry of health (MOH), Israel, CD4 and viral load (VL) trajectories

## Abstract

In Israel, irregular migrants (IMs) living with human immunodeficiency virus (HIV-1) that have no access to regular health insurance are provided with HIV-1-related health coverage under a public–private partnership (PPP) program initiated by the Ministry of Health in 2014. Here we characterized IMs referred to the PPP in 2019–2024 and used a linear mixed-effects model to follow up their CD4 and HIV-1 viral load (VL) counts for a median period of 16 months. Subtypes, resistance mutations and phylogenetic relationships were determined in all cases with viral failure and in selected cases with available blood remains. A total of 231 of 238 referred to the PPP initiated antiretroviral treatment (ART) with nucleoside reverse transcriptase inhibitors (NRTIs) and either non-nucleoside reverse transcriptase inhibitors (NNRTIs, 61.5%, 142/231) or protease inhibitors (PIs, 38.5%, 89/231). Irrespective of the treatment regimen, all these individuals increased their CD4 and decreased their VL trajectories over time (*p* < 0.001). However, mixed model analysis revealed two classes of CD4 trajectory patterns. Comparison between these two patterns revealed that Class-1 individuals started with lower initial CD4 counts compared to Class-2 individuals (median of 115 cells/mm^3^, IQR 70–171 compared to median of 312 cells/mm^3^, IQR 104–510, *p* < 0.001) and experienced slower recovery compared to Class-2. Most Class-1 individuals originated from Africa (78% vs. 52%, *p* = 0.016). Treatment failure was observed in 5.6% of all individuals receiving treatment under the program. Sequencing analysis, enabled in 23% of the treated cohort, revealed that the reverse transcriptases (RT) M184V (13%) and K103N (7.4%) were the most prevalent mutations. Conclusively, while treatment was not consistent with current recommendations for first-line therapy, the virological and immunological response of most patients was favorable and the prevalence of cases with resistance mutations was not higher than that identified in people living with HIV-1 who are covered by the national health insurance. Despite the limitations of the PPP, this program may provide a unique therapeutic opportunity for IMs with HIV-1.

## 1. Introduction

Many of the newly identified people living with HIV-1 (PLHIV) in developed countries are migrants, including those arriving from countries characterized by high human immunodeficiency virus (HIV) prevalence [1]. Upon arrival, these migrants may not have full access to HIV prevention, testing and treatment services [2,3]. Among all such migrants, irregular migrants (IMs) are the least likely to have access to care, as they usually do not have medical insurance [4,5]. Inadequate access to antiretroviral therapy (ART) is known to result in higher risk for ongoing transmission [6] and the development of comorbidities [7].

Israel is a country of ~10 million citizens and hosts [8], including ~111,000 (1.2%) foreign laborers with a valid working visa and ~25,000 (0.3%) IMs [9]. IMs living with HIV-1 are not covered by medical insurance and do not have free access to public health-care services [10], in contrast to all citizens and labor migrants who hold a valid working visa.

To provide appropriate care for such IMs, the Ministry of Health (MoH), together with pharmaceutical companies and the acquired immune deficiency syndrome (AIDS) Task Force (the largest nongovernmental organization in this field), initiated a public–private partnership (PPP) program in 2014 [11]. Those eligible for the program are IMs who are living with HIV/AIDS, have arrived in Israel at least 6 months before referral to the program (to prevent health tourism), have an official form of identification (asylum application, visa or passport), are ineligible for national insurance and lack private health insurance. A medical committee composed of HIV/AIDS experts determines the medical criteria required to include candidates in the program [11]. Antiretroviral treatment (ART), the guidelines for treatment initiation, the schedule of clinical follow-up visits and the availability of monitoring tests are set by the program [10].

This study aims to characterize all IMs who are PLHIV that were referred to the program between January 2019 and April 2024 and evaluate the time-related efficacy based on clinical and virological parameters by the integration of trajectory modeling of CD4 recovery. Molecular epidemiology and resistance analysis were also incorporated in a subset of cases with available and adequate sample remains.

## 2. Materials and Methods

### 2.1. Study Cohort and Data Collection

A retrospective, multisite, longitudinal cohort study was conducted on individuals referred to the PPP between January 2019 and April 2024. Data, stored in medical records, were collected routinely from regional HIV/AIDS clinics and cross-matched with the database of the National HIV Reference Laboratory (NHRL). IMs who received ART (see following section) were followed up until 2024 or until when they were naturalized and granted citizenship, left Israel or died.

Basic parameters including demographic (sex, age, country of birth, mode of HIV-1 transmission), virological (initial CD4 cells/mm^3^ and viral load (VL) log copies/mL at PPP referral) and clinical (pregnancy status, AIDS-defining diseases at PPP referral [12]) data were collected. CD4 counts were measured by flow cytometry and HIV-1 plasma viral loads were determined by an Xpert HIV-1 viral load assay [13] with a detection limit of 40 copies/mL.

Longitudinal data including ART regimens, CD4 counts and VL were also collected and recorded, as were HIV-1 subtypes and drug resistance mutation (DRM) from available nucleotide sequences.

Viral failure (VF) was defined as a confirmed increase in VL (either two times ≥ 50 copies/mL, as recommended in the European guidelines [14]) or as one or more consecutive measurements exceeding 200 copies/mL (as suggested by the American guidelines) [15].

The country of birth was categorized by region (details in the Supplementary Materials section), and the main modes of HIV-1 transmission were categorized as heterosexual (Hetero), men who have sex with men (MSM) and intravenous drug users (IVDUs).

### 2.2. Diagnosis, Monitoring and ART Under the PPP

As part of an initiative to improve access to HIV diagnosis for IMs, MoH offers free HIV-1 testing at numerous sites across Israel. To be included in the PPP, IMs with HIV-1 had to be either pregnant women or to have CD4 counts less than 500 cells/mm^3^ [11]. Monitoring under the PPP included annual viral load and CD4 testing with up to two instances of VL testing enabled in the first year after entering the PPP. Regarding ART, due to budgetary and logistical constraints, the options under the PPP were the protease inhibitors (PIs) atazanavir (ATZ), ritonavir (RTV) and lopinavir (LPV); the nucleoside reverse transcriptase inhibitors (NRTIs) tenofovir disoproxil fumarate (TDF), lamivudine (3TC), emtricitabine (FTC) and zidovudine (ZDV); and the non-nucleoside reverse transcriptase inhibitors (NNRTIs) efavirenz (EFV) and nevirapine (NVP). In 2020, the program introduced a single-tablet regimen (STR) consisting of EFV, TDF and FTC, aiming to simplify treatment and increase compliance [16]. During the study period (2019–2024), the PPP did not offer integrase inhibitors (INSTIs); therefore, the impact of more inferior regimens was assessed in the current study. Resistance testing under the PPP was approved in all cases of viral failure.

### 2.3. Study Participants

**Figure 1:**
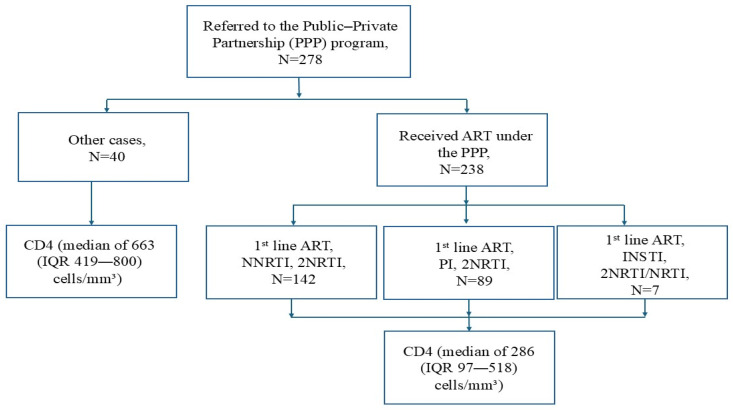
Flow diagram of the IMs living with HIV/AIDS referred to the program between 2019 and 2024. ART—antiretroviral therapy; IQR—interquartile range; NNRTIs—non-nucleoside reverse transcriptase inhibitors; NRTIs—nucleoside reverse transcriptase inhibitors; PIs—protease inhibitors; INSTIs—integrase strand transfer inhibitors.

A total of 278 IMs living with HIV/AIDS who were referred to the program between 2019 and 2024 from all HIV-1 regional clinical centers in Israel were initially assessed in this study. Only 238 of them, who were either pregnant women or with CD4 counts below 500 cells/mm^3^ and eligible for ART (according to the PPP guidelines), are included in this study. All other individuals (n = 40) with higher CD4 counts did not receive treatment under the PPP and were excluded from further analysis. As expected, those who were eligible for ART presented lower median levels of CD4 (286 cells/mm^3^, interquartile range, IQR, 97–518) compared to those who did not receive ART (663 cells/mm^3^, IQR 419–800, *p* < 0.001; Figure 1).

### 2.4. Sequencing, DRM Analysis and Genotyping

For sequencing, plasma HIV-1 RNA or whole blood DNA (in cases with viral load below 500 c/mL) was extracted and sequenced using Sanger population sequencing as previously described [17].

DRMs were determined in fragments obtained by polymerase chain reaction (PCR) amplification from the protease (HXB2 nucleotide PR; positions 2261–2549), reverse transcriptase (RT; positions 2660–3290) and integrase (IN; positions 4230–5093) genes. The Stanford Database HIVdb algorithm, version 9.6 [18,19], which returns inferred levels of resistance to 25 antiviral drugs and is regularly updated to incorporate new resistance patterns, was used for HIV-1 resistance interpretation (available at https://hivdb.stanford.edu/pages/surveillance.html, accessed on 10 October 2024). The Stanford database in HIVdb [18] and the Recombinant Identification Program (RIP, available at https://www.hiv.lanl.gov/content/sequence/RIP/RIP.html, accessed on 10 October 2024) were utilized to define classical subtypes and identify HIV-1 recombinants.

### 2.5. Phylogenetic Analysis

Partial protease and reverse transcriptase sequences were assembled via R script. Sequences shorter than 918 nucleotides were excluded. The resulting assembled HIV-1 sequences were aligned (using Ali View version 1.27) [20] with 142 additional representative reference sequences covering all known circulating recombinant forms (CRFs) and non-CRFs of group M, and 1 sequence of group O was used as the outgroup. These data were obtained from the Compendium of the Los Alamos database (https://www.hiv.lanl.gov/content/sequence/NEWALIGN/align.html (accessed on 10 October 2024)).

The maximum-likelihood phylogenetic method and the general time-reversible model were used for phylogenetic analysis (Mega X software, [21] with the proportion of invariable sites and gamma plus invariant sites-distributed rate heterogeneity (GTR + G + I model) based on the results of JModelTest [22] version 2.1.9. A maximum likelihood phylogenetic tree was constructed with branch support assessed from 1000 bootstrap replicates and visualized with Fig Tree version 1.4.4 (http://tree.bio.ed.ac.uk/software/figtree/, accessed on 10 October 2024).

### 2.6. Statistical Analysis and Ethical Approval

Statistical analysis was performed via R studio version 1.3.1093. The libraries used in this analysis included “gt”, “gtsummary”, “dplyr”, “ggplot2”, “tidyverse” and” lcmm”. For categorical data, chi-square or Fisher’s exact tests were used, and Wilcoxon rank sum tests were used to compare continuous variables with several distributions. Bonferroni correction was performed for multiple comparisons to avoid the risk of type 1 errors.

Linear univariate mixed-effects models with random intercepts and random slopes were employed to derive longitudinal CD4 (square root counts) and VL (log c/mL) trajectories [23]. The patterns of the CD4 and VL trajectories (intercepts and slopes) were determined according to the coefficients (random effects of negative/positive intercept and negative/positive slopes), which define a negative or positive effect on the longitudinal changes in the CD4 square root counts and log VL for each participant.

A sensitivity analysis of missing CD4 and VL test data was performed to impute missing values using mean extrapolation or interpolation (details in Supplementary Materials section).

Approval for this study was granted by the Sheba Institutional Review Board, IRB (SMC-D-1502-24). The need for consent to participate was waived by the Sheba IRB.

## 3. Results

### 3.1. Epidemiology

A total of 238 IMs started treatment in the PPP program during the study period. Among those, 117 (49%) were females and 15 (13%) were pregnant at the time of referral. The majority (72%) were heterosexuals. Most IMs (55%) originated from Africa, and 34% originated from Eastern Europe and Central Asia (EEU/CA) (Table 1).

Only 21% were initially diagnosed with HIV-1 in Israel. Most IMs (60%, 143/238) were diagnosed before arrival in Israel and the majority of the latter (84%, 120/143) were previously exposed to ART. While the majority of IMs that were first diagnosed in Israel were immigrants from African countries (67%, 34/51), those from EEU/CA were most commonly already diagnosed (45.2%, 47/104), and the majority (41/47 of those with known status) had previously been treated in their country of origin. Only 42% had CD4 ≥ 350 cells/mm^3^ and 20% had AIDS-defining conditions. Upon referral to the program, newly diagnosed individuals had significantly lower levels of CD4 (median of 115 cells/mm^3^) and higher VL (median of 5.12 log copies/mL) than those already known to be infected with HIV-1 (median CD4 233–462 cells/mm^3^ and VL 1.59–3.28 log copies/mL, *p* < 0.001). In general, males had lower median CD4 counts than females (189 cells/mm^3^, IQR 71–460, versus 364 cells/mm^3^, IQR 168–570, *p* = 0.001) and higher median levels of HIV-1 VL (4.14 log copies/mL, IQR 1.59–5.35 versus 2.39 log copies/mL, IQR 1.46–4.61, respectively, *p* = 0.033).

**Figure 2:**
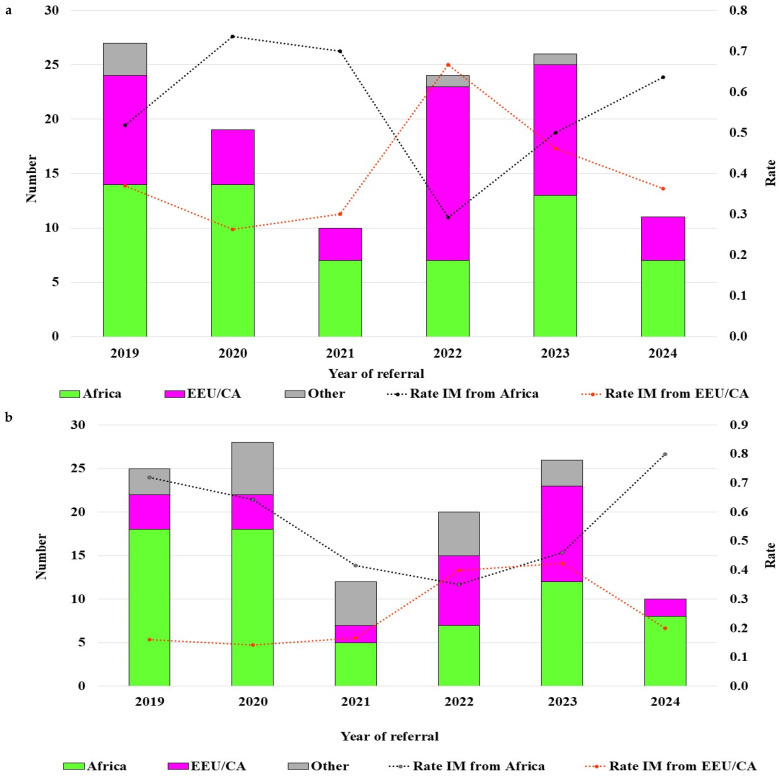
The number and relative proportion of IMs: ((**a**)—females; (**b**)—males) according to region of origin between 2019 (January) and 2024 (April). EEU/CA—Eastern Europe and Central Asia; IMs—irregular migrants.

When years of inclusion in the PPP were compared, an overall increase in the proportion of IMs from EEU/CA between 2019 and 2024 was observed (Figure 2a,b, trend, *p* = 0.1) with a peak in the number of female immigrants who left Russia and Ukraine in 2022 during the war between these countries (Figure 2a).

### 3.2. Follow-Up Analysis

Follow-up analysis was enabled in 231 of the 238 IMs in this study. Those seven individuals who started therapy in 2024, with regimens that included INSTIs, were excluded from the follow-up analysis.

During the median follow-up period of 16 months (IQR 7–34), 53% of them (123/231) switched ART at least once (Table S1), mainly due to unpleasant side effects and the logistic challenge of the programs. The most common first-line PPP regimens were NNRTI combined with two NRTIs (61.5%, 142/231) or PI combined with two NRTIs (38.5%, 89/231). The most common NNRTI-based regimen was EFV in combination with ZDV and 3TC, accounting for 73/142 (51.4%) of the cases. The most common PI was LPV/RPV in combination with TDF/FTC (64/89, 71.9% of cases). We detected no significant differences in baseline parameters, number of follows-up switches, or prevalence of VF between IMs who started ART with NNRTI plus two NRTI regimens and those who started PI with two NRTI regimens (Table S1).

**Figure 3:**
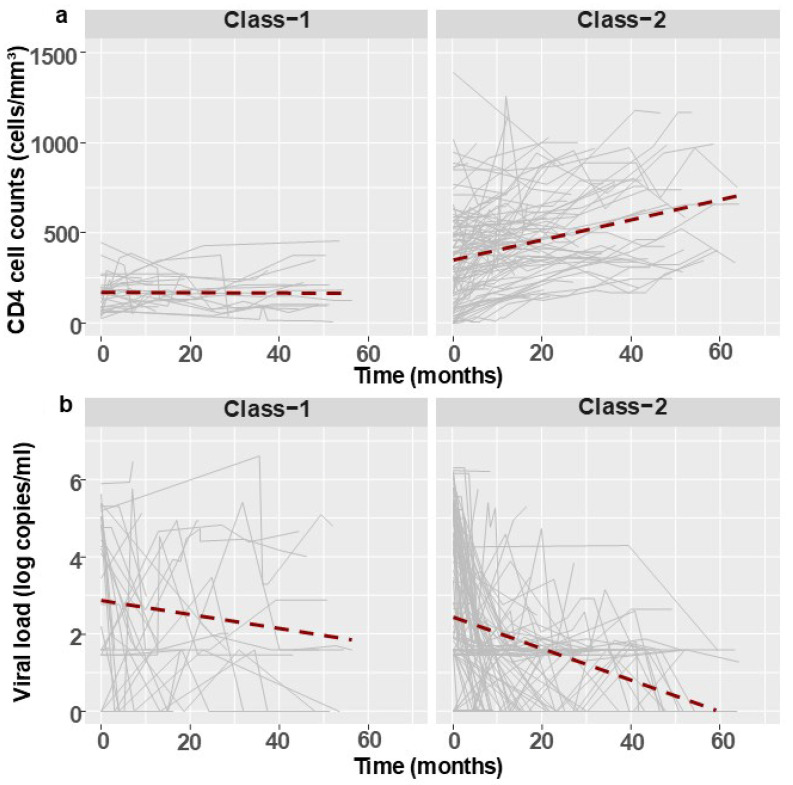
CD4 (**a**) and VL (**b**) trajectories by class (Class-1 and Class-2) over time in months. The grey color represents the individual CD4 and VL trajectories. The red line represents the linear trend line of the CD4 and VL trajectories.

To assess the effectiveness of ART in the PPP program, a univariate linear mixed-effects model was constructed using data from all IMs who had annual CD4 and VL results (in accordance with the program’s regulations). The analysis included 124 individuals, 62% (77/124) of whom initiated NRTI with NNRTI-based therapy and 38% (47/124) who started with NRTI and PI-based regimens. Overall, patients exhibited a significant increase in the CD4 trajectory (*p* < 0.001) and a decrease in the VL trajectory (*p* < 0.001) irrespective of the initial treatment regimen. Two classes of CD4 trajectories could be defined. Class-1 CD4 trajectories (27/124, 21.8% of patients) included individuals who had a gradual increase in CD4 counts whereas Class-2 CD4 trajectories (97/124, 78.2%) had a more rapid increase in CD4 levels (Figure 3a). In those individuals, a concomitant increase or decrease in HIV-1 VL was observed (*p* = 0.01, Figure 3b).

When the characteristics of individuals belonging to Class-1 CD4 trajectories were compared to those belonging to Class-2 CD4, we observed that the former had lower CD4 counts upon referral to the program (115 cells/mm^3^ median (IQR 70–171) vs. 312 cells/mm^3^ median (IQR 104–510), *p* < 0.001) and more of them failed therapy compared to Class-2 (7/27, 26% versus 3/97, 3.1%, *p* < 0.001), or originated from Africa (21/27, 78% vs. 50/97, 52%, *p* = 0.016; Table S2(a) in the Supplementary Materials section).

A sensitivity analysis conducted to evaluate the effect of missing data on these results confirmed the findings and classifications reported above (Tables S2 and S3, detailed in Supplementary Materials section).

### 3.3. Resistance Mutations, Subtyping and Phylogenetic Analysis

Overall, genotyping test (GT) results were available for 23.4% of the IMs that were followed up (54/231). Resistance testing following VF was requested for all 13 individuals (5.6%) who failed therapy (Table S4(a)). In all other cases (41/231), resistance testing was performed in available, pretreated samples, aiming to obtain more information on the viral subtype and resistance mutations in this cohort of IMs who are PLHIV. For these 41 individuals, sequencing was performed on pretreatment samples, irrespective of the initial therapy administered under the PPP (Table S4(b)).

In those who failed therapy, the median VL at VF was 5.08 log copies/mL (IQR 4.17–5.67), and the median time to treatment failure was 7 months (IQR 3–10). Patients who failed ART were most commonly on EFV-based ART (9/13, 69.2%, Table S4(a)). The majority of those who failed therapy were IMs from African countries (11/13, 84.6% vs. 21/41, 51.2%, *p* = 0.003) and half were within the Class-1 CD4 trajectories (7/13, 54% vs. 4/41, 9.8%, *p* = 0.013).

**Figure 4:**
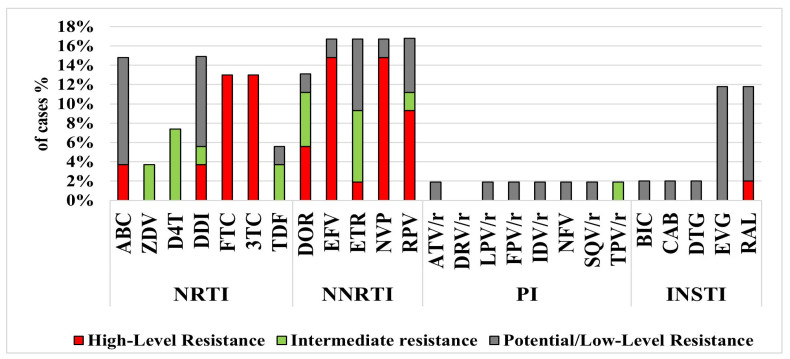
Proportion of the predicted drug susceptibility of all sequenced cases to different antiretroviral drugs. NRTIs—nucleoside reverse transcriptase inhibitors; NNRTIs—non-nucleoside reverse transcriptase inhibitors; PIs—protease inhibitors; INSTIs—integrase inhibitors; ABC—abacavir; ZDV—zidovudine; D4T—stavudine; DDI—didanosine; FTC/3TC—emtricitabine/lamivudine; TDF—tenofovir disoproxil fumarate; DOR—doravirine; EFV—efavirenz; ETR—etravirine; NVP—nevirapine; RPV—rilpivirine; ATV/r—atazanavir/ritonavir; DRV/r—darunavir/ritonavir; LPV/r—lopinavir/ritonavir; FPV/r—fosamprenavir/ritonavir; IDV/r—indinavir/ritonavir; NFV—nelfinavir; SQV/r—saquinavir/ritonavir; TPV/r—tipranavir/ritonavir; BIC—bictegravir; CAB—cabotegravir; DTG—dolutegravir; EVG—elvitegravir; RAL—raltegravir. The red color represents the proportion of cases with high-level resistance, the green color represents the proportion of those with intermediate-level resistance and the grey color represents the proportion of those with low/potential resistance.

PR and RT DRMs were identified in 16/54 of all sequenced cases: in 69% (9/13) of VFs compared to only in 17% (7/41) of the remaining sequences, *p* < 0.001 (Tables S4(a) and S5); DRM to PI, NRTI, and NNRTI mutations were identified in 7.7%, 38%, and 54% of patients with VFs, respectively. Overall, M184V (13%, 7/54) was the most common NRTI mutation, and K103N (7.4%, 4/54) was the most common NNRTI mutation (Tables S4 and S5). Multidrug resistance was observed in 15% (8/54) of the sequences, affecting mainly NNRTI (8/8) and one or two or more classes of ARTs (Tables S4 and S5). Figure 4 shows that DRM affected mostly susceptibility to the NNRTIs EFV and NVP; 14.8% of all sequenced cases presented high-level resistance. Additionally, high-level resistance to the NRTI FTC/3TC was observed in 13% of the cases.

**Figure 5:**
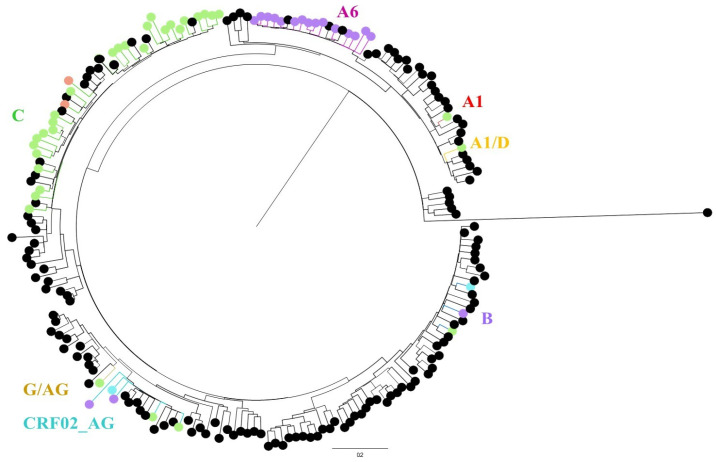
Phylogenetic analysis. The phylogenetic tree of the pol region of HIV-1 from 54 patients was generated via MEGA X and constructed via maximum likelihood (ML) with the GTR + G + I model. The 143 reference sequences (pure and recombinant) obtained from the Los Alamos database were also included. The tree was visualized in Fig Tree version 1.4.4. The HIV-1 subtypes are colored as follows: A1 in red, A1/D in orange, A6 in violet, C in green, G/AG in brown, CRF02_AG in turquoise and B in royal blue. The colored circles represent the place of birth as follows: Africa in green, EEU/CA in purple, Latin America in red and Palestine in blue. The reference sequences are in black. No clusters or connections between individuals were identified according to the bootstrap values (>0.8) and number of individuals (>2).

Phylogenetic (Figure 5) and demographic analysis revealed that subtype C, which characterized 52% of the available sequences, was identified primarily among migrants from Africa (26/32, 81.3%). On the other hand, subtype A6, which was identified in 27.8% of the sequences, was common among migrants from EEU/CA (15/18, 83%, *p* < 0.001). A total of seven recombinants (representing 13% of the sequences) were identified: A1/D (N = 1), CRF02_AG (N = 5) and G/AG (N = 1). No clusters or connections between sequenced cases were identified, suggesting that transmission within these random cases was unlikely.

## 4. Discussion

The present study focused on the characteristics and follow-up analysis of IMs who were referred to the PPP program during 2019–2024. In these years, the PPP ART regimens were not as advanced as regular clinical guidelines and did not include second-generation ARTs [14,15]. Furthermore, INSTIs were not introduced into the program before 2024. Despite these limitations, the longitudinal CD4 counts increased while HIV-1 VL measurements decreased over time, regardless of the specific treatment regimen. A subgroup consisting of 22% (27/124) of patients, mostly those who initially had lower CD4 cell counts, exhibited only a gradual increase in CD4 levels. Similar data have been reported previously for migrants who were treated in Germany and initiated their ART at a relatively later stage of HIV-1 infection compared to citizens [24]. These findings are consistent with the accumulating evidence that initiating ART in individuals with a CD4 count lower than 200 cells/mm^3^ may blunt immune response [25].

VF was recorded in only 5.6% (13/231) of the treated cases, similar to the proportion of VF observed in PLHIV who are Israeli citizens [26]. This result, which is surprising, may be attributed to the exceptionally high adherence of IMs to the program, ensuring reasonably good outcomes despite the inferior regimens provided by the PPP. Resistance testing was requested in all cases of VFs, whereas pretreatment resistance testing results were enabled only in a minority of the IMs (18.2%, 41/231). In most of these cases (27/41), a viral load >1000 copies/mL enabled sequencing from plasma samples. Pretreatment resistance testing was not mandatory in the PPP program because of the high cost of this test, which is estimated to be approximately USD 1500 [27], and because many individuals included in the program are already on some type of ART, with undetectable or very low HIV-1 VL. Nevertheless, the proportion of pretreatment DRMs identified here was similar to the proportion of pretreatment DRMs identified in treatment-naïve Israeli citizens (~20%), individuals for whom resistance testing at baseline is recommended and covered by the national insurance program [26,28].

Overall, mutations to NNRTI were the most common DRM (20%), of which K103N (which reduces susceptibility to several NNRTI including EFV) was the most prominent (7.4%). This may be attributed to the fact that 60% of the IMs in the PPP were previously treated in the country of origin with NNRTIs. However, HIV-1 drug resistance and surveillance data from countries in many resource-limited settings are thus far unavailable [29]. Additionally, NNRTIs, specifically K103N, were the most common mutations reported in seroconverted women from South Africa [30]. M184V was the most common NRTI mutation. This mutation is clinically relevant for all IMs in the PPP, as it reduces susceptibility to FTC and may affect several PPP regimens that include this drug. Additionally, M184V decreases susceptibility to 3TC [18,19] and is associated with an increased risk of developing resistance to dolutegravir (DTG) [31] in patients treated with the DTG/3TC two-drug regimen. Therefore, M184V should be continuously monitored, especially as this INSTI-based dual therapy has been included in the PPP since April 2024.

In general, when possible, pretreatment sequencing of the regions in HIV-1 that are targets for ART (PR, RT, IN, envelope, capsid) should be considered. Although not all DRMs are currently relevant in the context of the ART included in the PPP framework, in the future, they may become relevant following either changes in the regimens included in the PPP or acquisition of citizenship status by the IM, which will enable broader ART choices.

A larger proportion of IMs from EEU/CA, especially women, were referred to the PPP between 2019 and 2024 compared to previous period (2014–2018, 34% vs. 12.9%) [11]. Our analysis suggests that this increase could have resulted from the war between Russia and Ukraine [32]. Moreover, the flux of migration from Africa was reduced during the study period, probably partially due to COVID-19 and travel restrictions [33]. This trend was similarly observed across the European Economic Area, where the proportion of PLHIV originating from Eastern Europe increased from 8% to 23% of the total HIV-1 diagnoses [1,34].

The most common transmission mode was heterosexual contact, accounting for 98% of IM from Africa and 54% of those from EEU/CA. Indeed, an increase in the proportion of new HIV-1 infections from EEU/CA with a heterosexual mode of transmission has also been reported in Europe [34]. While in Israel, women with HIV-1 constitute ~30% of all citizens with HIV-1, 49% of the IMs were women. The majority (72%, 84/117) of the women referred to the PPP program were of childbearing age (≤45), and 13% of them (15/117, 13%) were pregnant at the time of referral. To minimize the risk of vertical transmission during pregnancy or childbirth, the Israeli MoH recommended universal HIV testing for all pregnant women [35]. All children of IMs can be medically insured if their parents pay an affordable premium to ensure full health coverage and eligibility for ART.

The most common (81.3%) HIV-1 subtype among IMs from Africa was subtype C, which has been identified in Israel in other groups of migrants from Africa [28,36]. In contrast, subtype A6, which has been dominant in the EEU/CA region for nearly three decades [28,36], was common (83%) among migrants from that region. The associations between viral subtypes, DRMs and VFs have already been demonstrated [37]. For example, IN gene mutations such as G140 and Q148 combined with the L74-mutated site, which is prominent in A6, could affect the potency of cabotegravir [38]. Therefore, determining the HIV-1 subtype and the identification of subtypes such as A6 may be imperative in the future when new therapies are introduced.

Despite the inherent difficulty in accurately assessing the origin of HIV-1 infection, the documentation indicates that at least 60% of individuals had been diagnosed with infection prior to their arrival in Israel. Indeed, phylogenetic analysis did not reveal prominent clusters or connections between individuals, suggesting that transmission within this cohort of random cases was unlikely. In order to provide a conclusion regarding the transmission of HIV-1 within the whole population of IMs who are PLHIV, a larger number of sequenced cases should be analyzed phylogenetically. Moreover, a follow-up study is needed to assess the future spread of HIV-1.

The relatively low CD4 counts (median of 286 cells/mm^3^) and the AIDS-defining conditions [12] upon referral to the PPP are alarming. Israeli-born PLHIV diagnosed between 2010 and 2018 had median CD4 counts of 400 cells/mm^3^ [28]. This difference may be related to different barriers to HIV-1 diagnosis among migrants as has been observed elsewhere [2,3,39], although HIV-1 testing in Israel is available to both citizens and IMs at numerous sites and is free of charge [11,40].

A review published in The Lancet Public Health in 2022 claims that universal healthcare coverage in Europe offers early multi-disease testing for HIV, as well as treatment, as part of an initiative integrated with existing health systems [41]. However, a different recent report [42] shows that only 15 of the 52 reporting countries across Europe and Central Asia actually provide free-of-charge treatment to undocumented migrants; 10 of these are in the west sub-region, 3 in the center and 2 in Eastern Europe. Indeed, Germany and Greece present different models of HIV care for IMs. While inclusive, government-supported healthcare policies in Germany may improve HIV treatment for IMs, restrictive policies in Greece may lead to treatment interruptions and an increase in HIV transmission [43]. The Israeli PPP described here, which is continuously monitored by the MoH, may provide a unique example of accessing ART for IMs in a high-income country, with its successful integration into the national health system.

Indeed, the European Network’s report that assessed the status of health inequalities in the European Union region found that only 9.2% (799/8696) of non-citizens in the European Economic Area have full healthcare coverage [44]. Many healthcare systems lack the infrastructure necessary to provide effective care to IMs. This deficiency is largely attributed to the absence of policies specifically tailored to address the needs of this population, the scarcity of cultural and linguistic facilitators to assist with communication and the lack of culturally and migrant-sensitive training programs available for healthcare providers [45]. One method that is practiced in Israel and aims to increase the linkage to care and to ensure timely periodic testing includes culturally competent HIV treatment centers, with dedicated social workers and trained community workers who provide emotional support, build trust and help to reduce the negative stigma.

This study had several limitations. First, not all IMs living with HIV were included in the PPP program. Pre-selection of individuals based on clinical parameters as well as exclusion of those IMs who were unable to reach referral services results in selection biases and may limit the generalization of the results. Also, the IMs’ care-seeking behaviors are most probably different from those of fully insured Israeli citizens [10]. Therefore, this study was performed separately from previous studies on citizens living with HIV-1 in Israel [26], resulting in a lack of a concurrent control group. Reporting biases may also be present for risk behaviors associated with HIV infections. Another limitation results from the fact that data on the place of infection were lacking for some individuals and therefore could not be fully analyzed. Also, resistance testing, which was performed in all cases of VF and in a subset of cases with available blood remains, was not performed in all the cases included herein. The use of Sanger sequencing with limited sensitivity to minority variants may also affect the rate and scope of DRMs identified. In order to increase sensitivity, it is recommended that the next-generation sequencing (NGS) technique be employed in future studies.

Another limitation is that longitudinal CD4 and VL results were available for only 52% of the patients. Furthermore, direct adherence measurements were not performed. This is due to the fact that drug blood levels are not monitored in Israel, and any attempt to assess them indirectly is problematic, particularly in IMs. This is mainly due to the differences in follow-up visits and the reduced availability of monitoring tests in IMs compared to those in Israeli citizens. Consequently, adherence measurements were not available.

Regardless of these limitations, our study demonstrates that the clinical outcome of IMs under the PPP is not significantly different from that of PLHIV who are covered by health insurance [26].

The strengths of this study are that it focused on a unique, hard-to-reach real-world cohort and implemented a longitudinal follow-up analysis involving mixed-effects modeling. Moreover, the integration of clinical and molecular data provided a comprehensive view of IMs under antiretroviral treatment in the years 2019–2024.

Early HIV-1 diagnosis and concomitant referral to the PPP program, together with ART and continuous monitoring, are pivotal stages in the adaptation of intervention strategies when aiming to improve the containment of the HIV-1 epidemic in Israel in general, as well as within the IM population.

## Figures and Tables

**Table 1:** Baseline characteristics of irregular migrants starting ART under the PPP from 2019 to 2024.

Characteristics	Overall, N = 238
Age at referral, median (IQR), years	40 (35–49)
HIV-1 status	
Previously known with HIV-1, n (%)	143 (60)
Previously known abroad	23 (23/143)
Previously known and treated abroad	81 (81/143)
Previously treated in Israel	39 (39/143)
Newly identified in Israel, n (%)	51 (21)
Previous status unknown, n (%)	44 (19)
Time from diagnosis to PPP referral, median (IQR), months	6 (6–12)
Sex	
Female, n (%)	117 (49)
Male, n (%)	121 (51)
Birthplace	
Africa, n (%)	130 (55)
EEU/CA, n (%)	81 (34)
Other, n (%)	27 (11)
Transmission	
Hetero from Africa, n (%)	128 (54)
Hetero from EEU/CA, n (%)	44 (18)
Hetero from other regions *, n (%)	8 (3.4)
MSM, n (%)	27 (11)
IVDU, n (%)	13 (5.5)
MTCT, n (%)	1 (0.4)
Unknown, n (%)	17 (7.1)
CD4 at referral, median (IQR), cells/mm^3^	286 (97–518)
CD4 by groups	
<200, n (%)	95 (40)
200–350, n (%)	42 (18)
≥350, n (%)	101 (42)
VL, (N = 190) at referral, median (IQR), log copies/mL	2.80 (1.51–5.09)
Pregnancy, (N = 117), n (%)	15 (13)
AIDS defining disease upon referral, n (%)	52 (22)

n—number; ART—antiretroviral therapy; IQR—interquartile range; VL—viral load; HIV-1—human immunodeficiency virus 1; EEU/CA—Eastern Europe and Central Asia; MSM—men who have sex with men; IVDUs—intravenous drug users; MTCT—mother-to-child transmission; AIDS—acquired immunodeficiency syndrome; PPP—public–private partnership; * Other regions: Thailand (N = 3), Philippines (N = 2), Colombia (N = 1), India (N = 1), Ecuador (N = 1).

## Data Availability

Raw data can be provided upon request from the authors.

## Supplementary Materials

The following supporting information can be downloaded at: https://www.mdpi.com/article/10.3390/v17121566/s1, Detailed method section and supplementary Tables S1–S5. Supplementary Table S1: Comparison between the characteristics of 231 individuals receiving NNRTI, 2NRTI versus PI, 2NRTI as first-line ART under the PPP program. Supplementary Table S2: Comparison between the characteristics of individuals in Class-1 and Class-2 CD4 trajectories (a): Results of the main model on 124 individuals. (b). Results of the model on 152 individuals. Supplementary Table S3: Comparison between the characteristics of individuals with complete data (annual CD4 and VL data) and those with partial data (incomplete imputed group) and those with single-point VL or CD4 data (dropout group). Supplementary Table S4: Characteristics and clinical data on those with VF (N = 13) (a) and on cases with pretreatment resistance results (N = 41) (b). Supplementary Table S5: Number and proportion of the most frequently detected DRMs according to HIV-1 subtypes.

## Abbreviations

The following abbreviations are used in this manuscript:

IMs	irregular migrants
HIV-1, HIV	human immunodeficiency virus
PPP	public–private partnership
VL	viral load
ART	antiretroviral treatment
NRTIs	nucleoside reverse transcriptase inhibitors
NNRTIs	non-nucleoside reverse transcriptase inhibitors
PIs	non-nucleoside reverse transcriptase inhibitors
RTs	non-nucleoside reverse transcriptase inhibitors
PLHIV	people living with HIV-1
MoH	people living with HIV-1
NHRL	National HIV Reference Laboratory
DRM	drug resistance mutation
VF	viral failure
Hetero	heterosexual
MSM	men who have sex with men
IVDUs	intravenous drug users
ATV	atazanavir
RTV	ritonavir
LPV	lopinavir
TDF	tenofovir disoproxil fumarate
3TC	lamivudine
FTC	emtricitabine
ZDV	zidovudine
EFV	efavirenz
NVP	nevirapine
STR	single tablet regimen
INSTIs	integrase inhibitors
PCR	polymerase chain reaction
PR	protease
RT	reverse transcriptase
IN	integrase
RIP	Recombinant Identification Program
CRFs	circulating recombinant forms
IRB	Institutional Review Board
EEU/CA	Eastern Europe and Central Asia
GTs	genotyping tests
US	United States
DTG	dolutegravir
NGS	next-generation sequencing
