# Supplementary material for: Accessing HIV Care to Irregular Migrants in Israel, 2019–2024
Source: Viruses. 2025 Nov 29;17(12):1566. doi: 10.3390/v17121566 (PMC12737707; doi:10.3390/v17121566)
Supplement: Supplementary file 1 [file viruses-17-01566-s001.zip › viruses-3971637-supplementary.pdf]

Supplementary Materials section:

Categorization of birthplace

Birthplace was categorized as follows which was based on https://aidsinfo.unaids.org/

definition: Africa (Ethiopia, Eritrea, Ghana, Sudan, South Africa, Kenya, Uganda, Botswana, Guinea, Zimbabwe, Ivory Coast, Lesotho, Malawi, Nigeria, Democratic Republic of the Congo), Eastern Europe and Central Asia, EEU/CA, (Russian Federation, Ukraine, Republic of Moldova, Belarus, Georgia), and other (France, Argentina, Costa Rica, Colombia, Ecuador, Venezuela, India, Philippine, Thailand, Taiwan, China, Palestine).

Table S1: Comparison between the characteristics of 231 individuals receiving NNRTI, 2NRTI versus PI, 2NRTI as first- line ART under the PPP program.

	NNRTI, 2NRTI N=142	PI, 2NRTI N=89	*p*-Value
CD4 at referral median (IQR), cells/mm³	263 (100-490)	342 (107-534)	0.5
HIV-1 RNA, N =184, n (79,7%)	N=110	N=74	
HIV-1-RNA, at referral, median (IQR), log copies/mL	3.37 (1.51-5.25)	2.78 (1.59-4.84)	0.7
CD4 Class			
Class -1, N=124, n (53.7)	17 (22)	10 (21)	>0.9
Class -2, N=124, n (53.7)	60 (78)	37 (79)	
HIV-1 Subtype, (N=54) **	N=33	N=21	
A6, n (%)	9 (27)	6 (29)	0.13
C, n (%)	20 (61)	8 (38)	
Other, n (%)	4 (12)	7 (33)	

PR-RT DRM by class (N = 54), n (%)	8 (24)	8 (38)	0.3
NNRTI, n (%)	6 (18)	5 (24)	0.7
NRTI, n (%)	4 (12)	6 (29)	0.2
PI, n (%)	1 (3)	0	>0.9
Switches (number)			
"0" switches, n (%)	71 (50)	37 (42)	0.1
"1" switch, n (%)	38 (27)	34 (38)	
"2" switches, n (%)	17 (12)	14 (16)	
">2" switches, n (%)	16 (11)	4 (4.5)	
Time on first-line ART, median (IQR), months	12 (4-25)	14 (5-23)	0.8
VF, n (%)	9 (6.3)	4 (4.5)	0.6

n-number; ART- antiretroviral therapy; IQR – Inter Quartile Range; HIV- Human Immunodeficiency Virus; ART- antiretroviral therapy; PPP- Public-Private Partnership; PR-protease; RT-reverse transcriptase; PI- protease inhibitors; NNRTI—non-nucleoside reverse transcriptase inhibitors; NRTI- nucleoside reverse transcriptase inhibitors; DRM – drug resistance mutations; VF-viral failure. Switches (number)- number of the regimen changes within the PPP.

Sensitivity analysis for missing data

A sensitivity analysis was conducted to evaluate the impact of missing data on the results reported based on the data from the group that had annual CD4 and VL data (complete group, N = 124). In the incomplete group (N=28) the available CD4 and VL results were used to impute the missing CD4 and VL results using mean extrapolation or interpolation. The main model was first run using the "complete" group only with HLME (), N = 124 and a total of 571 observations (https://doi.org/10.18637/jss.v078.i02). Next, the same model was rerun with the additional 28 cases of imputed data (N = 124 + 28 = 152 and a total of 675 observations). Even though the incomplete group was characterized with higher CD4 at PPP referral (356 cells/mm^3, IQR 233-264, versus 247 cells/mm^3,

IQR 84-477, $p<0.01$, Table S3) the very similar p value observed in both runs (Table S2) confirms the findings and classifications reported by the main model for the "complete" group only.

Supplementary Table S2a, b: Comparison between the characteristics of individuals in Class-1 and Class-2 CD4 trajectories between the complete group (main model, N=124, S2a) versus the additional imputed group (N=124+28, S2b).

a. Complete group (main model, N=124)					*b. Complete and incomplete imputed group (N=124+28)*			
Characteristics	Overall, N=124	Class-1, N=27	Class-2, N=97	*p*-Value	Overall, N=152	Class-1, N=34	Class-2, N=118	*p*-Value
Age at referral, median (IQR), years	40 (35-47)	41 (38-48)	40 (35-47)	0.4	40 (35-47)	41 (37-51)	40 (35-46)	0.1
Sex								
Female, n (%)	59 (48)	11 (41)	48 (49)	0.4	77 (51)	16 (47)	61 (52)	0.6
Male, n (%)	65 (52)	16 (59)	49 (51)		75 (49)	18 (53)	57 (48)	
Birthplace								
Africa, n (%)	72 (58)	21 (78)	51 (53)	**0.015**	85 (56)	25 (74)	60 (51)	**0.041**
EEU/CA, n (%)	35 (28)	6 (22)	29 (30)		48 (32)	8 (24)	40 (34)	
Other, n (%)	17 (14)	0	17 (18)		19 (13)	1 (2.9)	18 (15)	
Transmission								
Hetero from Africa, n (%)	71 (57)	21 (78)	50 (52)	**0.016**	84 (55)	25 (74)	59 (50)	**0.050**

Hetero from EEU/CA, n (%)	17 (14)	3 (11)	14 (14)		23 (15)	4 (12)	19 (16)	
Hetero from other regions*, n (%)	5 (4)	0	5 (5.2)		6 (3.9)	1 (2.9)	5 (4.2)	
MSM, n (%)	15 (12)	0	15 (15)		17 (11)	1 (2.9)	16 (14)	
IVDU, n (%)	6 (4.8)	3 (11)	3 (3.1)		9 (5.9)	3 (8.8)	6 (5.1)	
Unknown, n (%)	10 (8.1)	0	10(10)		13 (8.6)	0	13 (11)	
CD4 at referral median (IQR), cells/mm^3	247 (84-477)	115 (70-171)	312 (104-510)	**<0.001**	270 (102-490)	128 (70-205)	349 (156-534)	**<0.001**
HIV-1 RNA, N	(N=118)	(N=26)	(N=92)		(N=131)	(N=29)	(N=102)	
HIV-1-RNA, at referral, median (IQR), log copies/mL	3.6 (1.6-5.2)	4.4 (1.8-5.3)	3.4 (1.6-5.1)	0.4	3.4 (1.6-5.2)	4.2 (1.5-5.3)	2.9 (1.6-5.0)	0.8
AIDS defining disease upon referral, n (%)	29 (23)	10 (37)	19 (20)	0.1	32 (21)	10 (29)	22 (19)	0.2
First line regimens								

within the PPP								
NNRTI, 2NRTI, n (%)	78 (63)	17 (63)	60 (62)	>0.9	90 (59)	21 (62)	69 (58)	0.8
PI, 2NRTI, n (%)	46 (37)	10 (37)	37 (38)		62 (41)	13 (39)	49 (42)	
Switches (number) >2 versus ≤ 2	16 (13)	6 (22)	10 (10)	0.1	19 (13)	6 (18)	13 (11)	0.4
VF, n (%)	10 (8.1)	7 (26)	3 (3.1)	**<0.001**	11 (7.2)	7 (20.6)	4 (3.4)	**0.001**
HIV-1 Subtype, (N=31)					HIV-1 Subtype, (N=36)			
A6, n (%)	7 (23)	1 (9.1)	6 (30)	0.6	7 (19)	1 (8.3)	6 (25)	0.6
C, n (%)	17 (55)	7 (64)	10 (50)		21 (58)	8 (67)	13 (54)	
Other, n (%)	7 (23)	3 (27)	4 (20)		8 (22)	3 (25)	5 (21)	
PR-RT DRM by class), n (%)	12 (39)	6 (55)	6 (30)	0.3	12 (33)	6 (50)	6 (25)	0.2
NNRTI, n (%)	9 (29)	4 (36)	5 (25)	0.7	9 (25)	4 (33)	5 (21)	0.4
NRTI, n (%)	8 (26)	4 (36)	4 (20)	0.4	8 (22)	4 (33)	4 (17)	0.4
PI, n (%)	1 (3.2)	1 (9.1)	0	0.4	1 (2.8)	1 (8.3)	0	0.4

n-number; IQR – Inter Quartile Range; PPP- Public-Private Partnership; MOH- Ministry of Health; EEU/CA- Eastern Europe and Central Asia; MSM- Men who have sex with men; IVDU- Intra venous drug users; AIDS-acquired immunodeficiency syndrome; PI-protease inhibitors; NNRTI—non-nucleoside reverse transcriptase inhibitors; NRTI- nucleoside reverse transcriptase inhibitors; VF – Viral failure. Variables with p- *value* =< 0.05 are shown in bold. CD4 Classes 1 and 2 were defined based on the results of Heterogenous Linear Mixed Model with random slope and random intercept to model the individual trajectory of CD4 levels over time. Individuals with pattern of Class-1 not only started with CD4 below average but also decreased their CD4 counts over time (i.e. random effects of negative intercept and negative slopes). Those belonging to Class-2 either started with CD4 level above average or increased the CD4 levels over time (i.e. Random effects were either positive intercept or slope).

* Hetero from other regions- hetero not from Africa and not from EEU/CA.

The comparison between the baseline characteristics of the group of individuals with complete CD4 and VL data (complete group) to those with incomplete data which was imputed (incomplete imputed group) to those with not enough data (the dropout group) is demonstrated in Table S3 below.

The imputed incomplete group was characterized with higher CD4 referral values compared to the "complete group". All other parameters were similar between the 2 groups. Within the dropout group 34% (29/86) individuals had left the program due to naturalization or departure from the country. Also, a small number 4.7% (4/86) in the drop group have died. This group may introduce some bias; however, this bias could not be accounted for due to the lack of follow-up information.

Supplementary Table S3: Comparison between the characteristics of individuals with complete data (annual CD4 and VL data) and those with partial data (incomplete imputed group) and those with single-point VL or CD4 data (dropout group).

Characteristics	Overall, N=238	Complete group, N=124	Incomplete imputed group, N=28	Dropout group, N=86
Age at referral, median (IQR), years	40 (35-49)	40 (35-47)	40 (34-49)	40 (34-51)
Sex				
Female, n (%)	117 (49)	59 (48)	18 (64)	40 (47)
Male, n (%)	121 (51)	65 (52)	10 (36)	46 (53)
Birthplace				

Africa, n (%)	130 (55)	72 (58)	13 (46.4)	45 (52.3)
EEU/CA, n (%)	81 (34)	35 (28)	13 (46.4)	33 (38.4)
Other, n (%)	27 (11)	17 (14)	2 (7.2)	8 (9.3)
Transmission				
Hetero from Africa, n (%)	136 (57.1)	76 (61.3)	14 (50)	46 (53.5)
Hetero from EEU/CA, n (%)	44 (18.5)	17 (13.7)	6 (21.4)	21 (24.4)
MSM, n (%)	27 (11.3)	15 (12.1)	2 (7.1)	10 (11.6)
IVDU, n (%)	13 (5.5)	6 (4.8)	3 (10.7)	4 (4.7)
Unknown, n (%)	18 (7.6)	10 (8.1)	3 (10.7)	5 (5.8)
HIV-1 Subtype, (N=56) **				
A6, n (%)	15 (27)	7 (23)	0	8 (40)
C, n (%)	30 (54)	17 (55)	4 (80)	9 (45)
Other, n (%)	11 (20)	7 (23)	1 (20)	3 (15)
CD4 at referral median (IQR), cells/mm^3	286 (97-518)	247 (84-477) [A]	356 (233-564) [B]	315 (94-589)
HIV-1 RNA, N = 190, n (80%)	N=190	N=118	N=13	N=59

HIV-1-RNA, at referral, median (IQR), log copies/Ml	2.8 (1.5-5.1)	3.6 (1.6-5.2)	1.7 (1.5-3.0)	2.5 (1.5-5.0)
AIDS defining disease upon referral, n (%)	52 (22)	29 (23)	3 (11)	20 (23)
First line regimens within the PPP, N=231	N=231	N=124	N=28	N=79
NNRTI, 2NRTI, n (%)	142 (61)	78 (63)	12 (43)	52 (66)
PI, 2NRTI, n (%)	89 (41)	46 (37)	16 (57)	27 (34)
	N=56	N=31	N=5	N=20
PR-RT DRM by class (N = 56), n (%)	17 (30)	12 (39)	0	5 (25)
NNRTI, n (%)	12 (21)	9 (29)	0	3 (15)
NRTI, n (%)	10 (18)	8 (26)	0	2 (10)
PI, n (%)	2 (3.6)	1 (3.2)	0	1 (5)
Naturalized, n (%)	28 (12)	13 (10)	2 (7.1)	13 (15)
Left Israel, n (%)	25 (11)	8 (6.5) [C]	1 (3.6)	16 (19) [D]
Death, n (%)	7 (2.9)	3 (2.4)	0	4 (4.7)

Number of CD4 + VL tests median (IQR),	3 (1-5)	4 (3-6) [E]	4 (3-5)	1 (1-1) [F]

n-number; IQR – Inter Quartile Range; PPP- Public-Private Partnership; EEU/CA- Eastern Europe and Central Asia; MSM- Men who have sex with men; IVDU- Intra venous drug users; AIDS-acquired immunodeficiency syndrome; DRM- drug resistant mutations; PI- protease inhibitors; NNRTI—non-nucleoside reverse transcriptase inhibitors; NRTI- nucleoside reverse transcriptase inhibitors; VL – viral load

p-*Value* between A and B was 0.02; p-*Value* between C and D was <0.01; p-*Value* between E and F was <0.01; Comparison of all other variables results in p-*Values >0.05*.

Supplementary Table S4 (a, b): Characteristics and clinical data on those with VF (N=13) (a) and on cases with pretreatment resistance results (N=41) (b)

a.

PID	Birthplace	Subtype	Treatment history before current treatment in the PPP	Year of referral in PPP	Last treatment before resistance test	Resistance mutations at VF	VL * copies/ml	Treatment following resistance test	Comments
460	Botswana	C	Unknown	2019	1st line EFV, TDF, FTC	NNRTI-K103N, V106M	109->38800->39	2nd line ATV, RTV, TDF, FTC	Low compliance
471	South Africa	C	LPV/RTV/ATV, TDF, FTC	2020	1st line EFV, ZDV, 3TC	NNRTI-A98G, K101E, G190S; NRTI-M184V, T215Y	29->33220->29	2nd line LPV, RTV, TDF, FTC	

477	Eritrea	C	Naive	2020	1st line EFV, ZDV, 3TC	PI-V82L	154000->above 10^{6}->1930	2nd line EFV, TDF, FTC-STR	TB
481	Ghana	CRF02_AG	Unknown	2020	1st line EFV, ZDV, 3TC	None	2820->132295->Unk	Stopped PPP	AIDS; was naturalized
494	Ukraine	CRF02_AG	Unknown	2020	1st line ATV, RTV, TDF, FTC	NRTI-M184V; INSTI-L74I	423->8480->7570	2nd line EVG/C, TDF, FTC-STR	PCP; persistent viremia
497	South Africa	C	Known abroad as HIV-1	2020	1st line EFV, ZDV, 3TC	None	Unk->592838->39	2nd line LPV, RTV, TDF, FTC	
500	Ghana	G/CRF02_AG	Unknown	2020	1st line EFV, TDF, FTC	NNRTI-K103N, V179E, Y181C. NRTI-M184V, K65R, Y115F	57->203000->119	2nd line ATV, RTV, TDF, FTC	HBV- (best choice was inclusion TDF/FTC); Adverse effects (1st line treatment)
520	Ivory Coast	CRF02_AG	Previously treated	2021	1st line LPV, RTV, TDF, FTC	NNRTI-E138A	TND->225311->Unk	Stopped PPP	Cryptococcosis; Death;

539	Palestine	CRF02_AG	Unknown	2021	1st line EFV, ZDV, 3TC	None	Unk->238000->Unk	Stopped PPP	He was imprisoned and later left Israel.
562	Nigeria	C	Unknown	2022	1st line LPV, RTV, TDF, FTC	NNRTI-A98G, K101E, G190A; NRTI-M184V	56000->56673->39	2nd line EVG/C, TDF, FTC-STR	
604	South Africa	C	Previously treated	2022	1st line ATV, RTV, TDF, FTC	NNRTI-K103N;	Above 10^6-> above 10^6->285000	Stopped PPP	
624	Eritrea	C	Naive	2023	1st line EFV, TDF, FTC-STR	None	Unk->2226->TND	Continued the 1st line	TB
632	South Africa	C	Previously treated	2023	1st line EFV, TDF, FTC-STR	NNRTI-K101E, V108I, Y181C, G190A, M221Y;	Unk->569000->TND	2nd line EVG/C, TDF, FTC-STR	

						NRTI-K65R, M184V			

VF – viral failure; VL-viral load; PPP - Public-Private Partnership; PID – patient ID; TND-target not detected; Unknown – Unk; TB – tuberculosis; PCP – Pneumocystis Pneumonia; HBV – Hepatitis B virus; VF was defined as either ≥twice increase in viral load above 50 copies/ml (as recommended in the European guidelines) or as one or more consecutive measurements exceeding 200 copies/ml (as suggested by the American guidelines).*VL was referred to as value of the VL before resistance test->at the resistance test->after resistance test.

b.

**PID**	**Birthplace**	**Subtype**	**Treatment history before current treatment in the PPP**	**Year of referral to PPP**	**Treatment following resistance test**	**Resistance mutations**
406	Ukraine	A6	Previously treated	2019	1st line EFV, TDF, FTC	INSTI-G163R, L74I

408	Ukraine	A6	naïve	2019	1st line EFV, ZDV, 3TC	None
448	Colombia	C	EVG/C, TAF, FTC-STR	2019	1st line EFV, ZDV, 3TC	None
455	Ethiopia	C	naïve	2020	1st line EFV, ZDV, 3TC	None
469	Russia	A6	LPV, RTV, ABC, 3TC	2020	1st line EFV, ZDV, 3TC	NRTI-A62V; INSTI-L74I
482	Argentina	C	EFV, TDF, FTC-STR	2020	1st line EFV, ZDV, 3TC	None
495	Ukraine	A6	DTG, TDF, FTC	2020	1st line ATV, RTV, TDF, FTC	INSTI-L74I
496	Sudan	C	naïve	2020	1st line EFV, TDF, FTC	None
509	Georgia	A6	Unknown	2020	1st line EFV, ZDV, 3TC	INSTI-L74I
518	Sudan	A1/D	naïve	2020	1st line EFV, ZDV, 3TC	None
531	South Africa	C	BIC, TAF, FTC	2021	1st line ATV, RTV, TDF, FTC	INSTI-E157Q
542	Ukraine	A6	BIC, TAF, FTC	2020	1st line LPV, RTV, ZDV, 3TC	INSTI-L74I
543	South Africa	C	naïve	2021	1st line LPV, RTV, TDF, FTC	None

544	Eritrea	B	naïve	2022	1st line LPV, RTV, TDF, FTC	None
550	Georgia	A6	Unknown	2021	1st line LPV, RTV, TDF, FTC	NRTI-M184V; INSTI-L74I
553	Georgia	A6	naïve	2022	1st line ATV, RTV, TDF, FTC	INSTI-L74I
555	Kenia	A1	Unknown	2021	1st line LPV, RTV, TDF, FTC	None
556	Ukraine	CRF02_AG	naïve	2022	1st line LPV, RTV, TDF, FTC	NNRTI- V179E, Y188L; NRTI-D67N, T69D, T215L, K219Q; INSTI-L74I
566	Ukraine	A6	naïve	2022	1st line LPV, RTV, TDF, FTC	INSTI-L74I
586	South Africa	C	LPV, RTV, TDF, FTC	2022	1st line LPV, RTV, TDF, FTC	None
592	Palestine	B	Previously treated	2022	1st line LPV, RTV, TDF, FTC	None
629	South Africa	C	Previously treated	2023	1st line EFV, TDF, FTC-STR	None
630	Russia	A6	Previously treated	2023	1st line EFV, TDF, FTC-STR	INSTI-L74I

636	Russia	A6	naïve	2023	1st line EFV, TDF, FTC-STR	INSTI-L74I
637	Congo	C	BIC, TAF, FTC	2023	1st line EFV, TDF, FTC-STR	None
645	Russia	B	DRV, RTV, TDF, 3TC	2023	1st line LPV, RTV, TDF, FTC	None
646	Ukraine	A6-PR-RT; CRF02_AG-INSTI	Previously treated	2023	1st line EFV, TDF, FTC-STR	NNRTI-V179E; INSTI-E138T, Y143R
647	Russia	A6	naïve	2023	1st line EFV, TDF, FTC-STR	INSTI-L74I
648	South Africa	C	Unknown	2023	1st line EFV, TDF, FTC-STR	None
652	South Africa	C	Unknown	2023	1st line EFV, TDF, FTC-STR	None
655	South Africa	C	ATV, RTV, TDF, FTC	2023	1st line LPV, RTV, TDF, FTC	NNRTI-K103N, V108I, H221Y, M230L; NRTI-M184V
656	Eritrea	C	DTG, TDF, FTC	2023	1st line EFV, TDF, FTC-STR	None
659	Moldova	A6	naïve	2023	1st line LPV, RTV, TDF, FTC	NRTI-A62V; INSTI-L74I
660	Eritrea	C	DTG, TDF, FTC	2023	1st line LPV, RTV, TDF, FTC	None

662	Ethiopia	C	naïve	2023	1st line LPV, RTV, TDF, FTC	INSTI-E157Q;
663	South Africa	C	EFV, TDF, FTC-STR	2023	1st line EFV, TDF, FTC-STR	None
669	Eritrea	C	naïve	2024	1st line EFV, TDF, FTC-STR	None
703	Eritrea	C	naïve	2024	1st line EFV, TDF, FTC-STR	None
707	South Africa	C	DTG, TDF, FTC	2024	1st line EFV, TDF, FTC-STR	None
715	South Africa	C	DTG, TDF, FTC	2024	1st line EFV, TDF, FTC-STR	NNRTI-E138A; INSTI-Q95K
718	Ukraine	A6	Unknown	2024	1st line EFV, TDF, FTC-STR	INSTI-T97A, L74I

NRTI, nucleoside reverse transcriptase inhibitors; NNRTI, non-nucleoside transcriptase inhibitors; PI, protease inhibitors; INSTI – integrase strand transfer inhibitors; PR-protease; RT-reverse transcriptase

LPV-lopinavir; RTV-ritonavir; ATV-atazanavir; DRV-darunavir; EFV-efavirenz; FTC- emtricitabine; 3TC- lamivudine; TDF-tenofovir disoproxil fumarate; TAF-tenofovir alafenamide; ZDV-zidovudine; ABC-abacavir; BIC-bictegravir; DTG- dolutegravir; EVG/C- elvitegravir/cobitistat; STR-single tablet regimen; 2DR-two drug.

Supplementary Table S5: Number and proportion of the most frequently detected DRMs according to HIV-1 subtypes.

Drug class	DRM	All N= 54	A1 1	A6 15	B 3	C 28	A1/D 1	AG 5	G/AG 1	A1 vs C	A1 vs G/AG	A6 vs C	A6 vs AG	A6 vs G/AG	C vs AG	C vs G/ AG	AG vs G/AG
**PI, n (%)**	V82L	1(1.9)				1(3.6)											
**NRTI, n (%)**	A62V	2(3.7)		2(13.3)													
	K65R	2(3.7)				1(3.6)			1(100)							***<0.001***	
	D67N	1(1.9)						1(20)									
	T69D	1(1.9)						1(20)									
	Y115F	1(1.9)							1(100)								
	M184V	7(13)		1(6.7)		4(14.3)		1(20)	1(100)			*0.464*	*0.403*	***0.010***	*0. 747*	**0.029**	*0.157*
	T215LY	2(3.7)				1(3.6)		1(20)							*0. 164*		

	K219Q	1(1.9)						1(20)									
**NNRTI, n (%)**	A98G	2(3.7)				2(7.1)											
	K101E	3(5.6)				3(10.7)											
	K103N	4(7.4)				3(10.7)			1(100)							***0.012***	
	V106M	1(1.9)				1(3.6)											
	V108I	2 (3.7)				2 (7.1)											
	E138A	2 (3.7)				1(3.6)		1(20)							*0.164*		
	V179E	3(5.6)		1(6.7)				1(20)	1(100)				*0.403*	***0.010***			*0.157*
	Y181C	2(3.7)				1(3.6)			1(100)							***<0.001***	
	Y188L	1(1.9)						1(20)									
	G190AS	3(5.6)				3(10.7)											
	H221Y	2(3.7)				2(7.1)											
	M230IL	1 (1.9)				1 (3.6)											
**NNRTI/NRTI, n (%)**		6 (11.1)				4(14.3)		1(20)	1(100)						*0.747*	***0.029***	***0.157***
		**N= 51**	**A1 1**	**A6 14**	**B 3**	**C 26**	**A1/D 1**	**AG 5**	**G/AG 1**	**A1 vs C**	**A1 vs G/AG**	**A6 vs C**	**A6 vs AG**	**A6 vs G/AG**	**C vs AG**	**C vs G/AG**	**AG vs G/AG**
**INSTI, n (%)**																	
**Major**	E138T	1(2)						1(20)									
	Y143R	1(2)						1(20)									

**Major INSTI/NNRTI, n (%)**		1(2)						1(20)									
**Accessory**	L74I	15(29.4)		13(93)				2(40)					***0.015***				
	Q95K	1(2)				1(3.8)											
	T97A	1(2)		1(7.1)													
	E157Q	2(3.9)				2(7.7)											
	G163R	1(2)		1(7.1)													
**Accessory INSTI/NNRTI, n (%)**		1(2)				1(3.8)											

DRM- drug resistance mutations; NRTI, nucleoside reverse transcriptase inhibitors; NNRTI, non-nucleoside transcriptase inhibitors; PI, protease inhibitors; INSTI – integrase strand transfer inhibitors. Data are presented as n (%). Differences in proportions were measured using the chi-squared test. Empty cells, n=zero.
